# Bile Acids as Inducers of Protonophore and Ionophore Permeability of Biological and Artificial Membranes

**DOI:** 10.3390/membranes13050472

**Published:** 2023-04-28

**Authors:** Victor N. Samartsev, Ekaterina I. Khoroshavina, Evgeniya K. Pavlova, Mikhail V. Dubinin, Alena A. Semenova

**Affiliations:** Department of Biochemistry, Cell Biology and Microbiology, Mari State University, pl. Lenina 1, 424001 Yoshkar-Ola, Russia; katya_bs@mail.ru (E.I.K.); evgewa2010_2011@mail.ru (E.K.P.); dubinin1989@gmail.com (M.V.D.); sem_al.ru@mail.ru (A.A.S.)

**Keywords:** bile acids, membranes, mitochondria, liver, protonophore, ionophore, nonspecific permeability

## Abstract

It is now generally accepted that the role of bile acids in the organism is not limited to their participation in the process of food digestion. Indeed, bile acids are signaling molecules and being amphiphilic compounds, are also capable of modifying the properties of cell membranes and their organelles. This review is devoted to the analysis of data on the interaction of bile acids with biological and artificial membranes, in particular, their protonophore and ionophore effects. The effects of bile acids were analyzed depending on their physicochemical properties: namely the structure of their molecules, indicators of the hydrophobic–hydrophilic balance, and the critical micelle concentration. Particular attention is paid to the interaction of bile acids with the powerhouse of cells, the mitochondria. It is of note that bile acids, in addition to their protonophore and ionophore actions, can also induce Ca^2+^-dependent nonspecific permeability of the inner mitochondrial membrane. We consider the unique action of ursodeoxycholic acid as an inducer of potassium conductivity of the inner mitochondrial membrane. We also discuss a possible relationship between this K^+^ ionophore action of ursodeoxycholic acid and its therapeutic effects.

## 1. Introduction

Bile acids and their salts form the basis of bile in higher vertebrates and humans. As part of bile, they play an important role in the process of digestion of food; their main physiological function is to emulsify bile lipids, in particular cholesterol. Emulsification of lipids by bile acids facilitates the absorption of fat-soluble vitamins and calcium in the intestine, and the digestion of triglycerols. In addition, bile excretion is necessary to eliminate toxins, carcinogens, as well as drugs and their metabolites. Apart from cholesterol, other endogenous compounds and metabolic products, such as bilirubin and hormones, are also excreted along with bile (see reviews [1,2]).

Bile acids are well known to be effective antimicrobial agents preventing the growth of bacteria in the small intestine [1,3,4]. Currently, the role of bile acids as signaling molecules regulating various metabolic pathways in cells is also being considered (see reviews [2,5,6,7,8,9]).

Primary bile acids—chenodeoxycholic (CDCA) and cholic (CA)—are synthesized from cholesterol in parenchymal cells (hepatocytes) of the liver of mammals and humans. In rodents, due to alternative hydroxylation, the formation of other primary bile acids is also possible, in particular, α-, β-, and γ-muricholic acids. Secondary bile acids are formed from these bile acids in the intestine with the participation of bacteria—lithocholic acid (LCA)-from CDCA, deoxycholic acid (DCA)-from CA. Additionally, in the intestine, ursodeoxycholic acid (UDCA) is formed from CDCA by epimeration of the hydroxyl group at the seventh carbon atom of the steroid nucleus (transfer of the hydroxyl group from the α-surface to the β-surface). A full description of the primary bile acid synthesis pathways is beyond the scope of this review and is covered in detail in the excellent review articles [2,5,8,10,11,12]. Prior to secretion, bile acids are conjugated with taurine or glycine [10,11,13].

Bile acids are considered as endogenous detergents. In particular, when interacting with artificial and biological membranes in concentrations exceeding their critical micelle concentration, bile acids cause profound disturbances in their structure and function, up to their permeabilization and lysis. It is of note that hydrophobic bile acids are most effective in this case [14,15,16,17]. The protonophore [18,19] and ionophore [20,21,22] effects of bile acids are also known (see the next section for more details).

In cholestasis caused by a blockage of the outflow of bile from the liver, the cells of this organ and the blood show first of all the accumulation of primary bile acids-CDCA and CA [23,24]. At concentrations characteristic of cholestasis, hydrophobic bile acids can solubilize lipids of cell membranes of hepatocytes leading to their damage and, in particular, to the release of γ-glutamyl transpeptidase from cells, whose increase in serum levels is a diagnostic sign of cholestasis [25]. In the course of chronic cholestasis, there is a violation of calcium homeostasis in hepatocytes and the release of calcium ions from the main depots—the endoplasmic reticulum and mitochondria. In this case, monohydroxy bile acids primarily release Ca^2+^ from the endoplasmic reticulum in the liver [26].

It is well known that hydrophobic bile acids, both primary CDCA and secondary LCA and DCA, as well as their glycine and taurine conjugates, have pronounced cytotoxicity [27,28,29]. LCA has been shown to selectively cause cell death in some malignant neoplasms [27,30]. Unlike other bile acids, UDCA is considered as a drug in the treatment of liver diseases and some other pathologies [31,32,33].

Several types of cell death are known, differing both in morphological features and in biochemical mechanisms. However, in all cases, the central role in these processes is assigned to mitochondria, which integrate many intracellular signaling pathways leading to cell death [28,34,35,36]. It is now generally accepted that one of the links in cell death associated with mitochondria is the opening of a Ca^2+^-dependent mitochondrial permeability transition pore (MPT-pore) in the inner membrane of these organelles for ions and hydrophilic substances, whose mass does not exceed 1.5 kDa, along their concentration gradient (see reviews [37,38,39,40,41]).

Bile acids have a variety of effects on the mitochondria of vital mammalian organs; they inhibit electron transport along the respiratory chain, induce a decrease on state 3 respiration, the respiratory control ratio, and the membrane potential and cause the induction of the MPT-pore [42,43,44,45,46,47,48,49,50].

This review is devoted to the analysis of literature data on the interaction of bile acids with mitochondria associated with their protonophore and ionophore effects, as well as the induction of the Ca^2+^-dependent MPT-pore. Particular attention is paid to the analysis of the effects of bile acids depending on their physicochemical properties: the structure of their molecules, indicators of the hydrophobic–hydrophilic balance, and the critical micelle concentration.

## 2. Physico-Chemical Characteristics of Bile Acids

**Figure 1:**
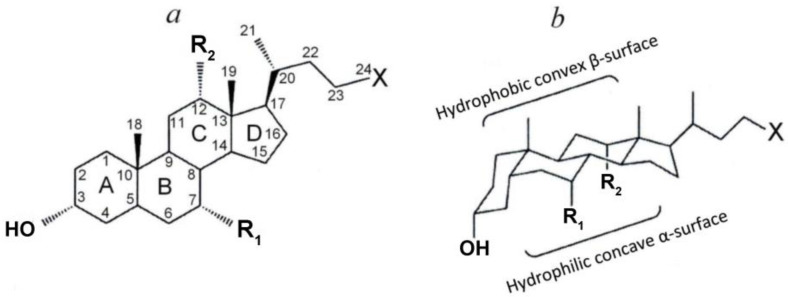
(**a**) Planar representation of the general molecular structure of bile acids. Letters (A, B, C, D), indicate the rings of the steroid skeleton, numbers—the carbon atoms, labels R_i_—the functional groups (hydroxyl groups or hydrogen atoms), and X—carboxyl group (non-conjugated bile acids) or their glycine and taurine conjugates. (**b**) Chair representation of the general molecular structure of BAs [1] with modifications.

Bile acids are a family of amphiphilic molecules containing a hydrophilic carboxyl group and a hydrophobic steroid structure (steroid core) with a different number of hydroxyl groups. The steroid core is considered as a flat structure with two surfaces depending on the localization of the hydroxyl groups—a more hydrophobic convex β-surface and a more hydrophilic concave α-surface (Figure 1). The whole variety of bile acids (Figure 1 and Table 1) is due to the different number and location of the hydroxyl groups of the steroid core, as well as their conjugation with taurine or glycine [1,11,13,16,51,52,53].

Free (non-conjugated) bile acids are weak acids (pKa of the carboxyl group in aqueous solution is 5.0). Conjugated bile acids are stronger acids, in particular, the pKa values for glycine conjugates in an aqueous solution are 3.9 for tauric ones—in the range from −1.5 to 1.5 [1,55,56,57]. Therefore, at physiological pH values (about 7.4), bile acids dissociate to form their salts. Bile salts are more water soluble than bile acids [52]. The pK_a_ values of the bile acids carboxyl group on the membrane surface is 2.5 units higher than in an aqueous solution [58]. Further, in all cases, we use the term “bile acids”.

Bile acids, being amphiphilic compounds, like other similar amphiphilic compounds, are detergents. However, bile acids differ significantly from classical detergents [13,16,59,60]. In classical amphiphiles, in particular lipids, the hydrophilic head consists of relatively small polar or charged groups, and the hydrophobic tails are often long, flexible, and non-polar hydrocarbon chains. Bile salts show other features. The polar hydroxyl groups are oriented towards the concave side of the rigid steroid ring system, which thus becomes hydrophilic, while the convex side is hydrophobic [1,13,16,59,60]. Thus, bile salts have a face structure with hydrophobic and hydrophilic sides or, depending on the position and orientation of the hydroxyl groups, a hydrophilic ‘edge’ only. Consequently, the hydrophilic and hydrophobic domains are not as clearly separated as is typical for classical amphiphiles. In addition, the hydrophobic tails of common amphiphiles inside the micellar core are liquid-like, while the steroid ring system is very rigid [1,13,16,59,60]. The hydrophobic–hydrophilic balance of bile acids as well as other amphiphilic compounds can be quantified as the partition coefficient between the lipid and aqueous phases (P or logP) (Table 1). The lower the value of this coefficient, the less hydrophobic and, therefore, more hydrophilic is the bile acid. As can be seen from the table, the values of this coefficient differ significantly depending on the method of their determination. However, depending on the structure of the bile acid molecule (the number of hydroxyl groups), the values of these coefficients show that monohydroxyl LCA is the most hydrophobic, dihydroxyl DCA, CDCA, and UDCA are less hydrophobic, and trihydroxyl cholic acid is the least hydrophobic. As follows from the data in the table, the hydrophobicity of glycine and taurine conjugates is significantly less than the corresponding non-conjugated acids.

Like amphiphilic compounds, bile acids are capable of self-organization with the formation of micelles when their concentration in water is increased to a certain level [13,52,53,61]. This extreme concentration of bile acids is called the critical micelle concentration (CMC) [13,52,53,60]. CMC values for bile acids and their glycine and taurine conjugates are shown in Table 1. Bile acids can form either primary or secondary micelles. Primary bile acid micelles have aggregation numbers from 2 to 10 and are formed via hydrophobic interactions, while secondary micelles (aggregation numbers 10–100) are formed via hydrogen bonding interactions of the primary micellar structures [13,59,60]. The pH at which CMC formation occurs is called the critical micellar pH at which the solubility increases markedly [52]. The CMC of trihydroxy bile acids is greater than the CMC of dihydroxy bile acids, and this value in turn is greater than the CMC of monohydroxy LCA (Table 1). The high CMC of trihydroxy bile acids is ascribed to their higher solubility in water [52,59]. It has been reported that conjugation with glycine or taurine slightly lowers the CMC of bile acids (Table 1) [53]. The orientation of hydroxy substituents also influences the CMC values and the changing of a hydroxy substituent from α- to β-configuration increases the CMC values. Moreover, addition of Na^+^ to a total concentration of 0.15 M lowers the CMC [53]. A more detailed consideration of the structure and kinetics of micelle formation by bile acids is beyond the scope of this review and is covered in detail in the original articles [61,62,63,64] and excellent review articles [1,13,59,60,65,66,67].

## 3. Effect of Bile Acids on the Permeability of Biological and Artificial Phospholipid Membranes for Protons and Ions

### 3.1. Protonophore Action of Bile Acids

As mentioned above, free (non-conjugated) bile acids are weak acids. A large number of weak organic acids are known to be effective protonophore uncouplers of oxidative phosphorylation in animal mitochondria [68,69,70,71]. Among them, the mechanism of the uncoupling action of classical protonophore uncouplers (2,4-dinitrophenol or DNP, carbonyl cyanide 4-(trifluoromethoxy)phenylhydrazone, or FCCP, etc.) has been well studied. These uncouplers, by increasing the proton conductivity of the inner mitochondrial membrane and thus dissipating the proton motive force (Δ*p*), stimulate respiration and reduce the efficiency of oxidative ATP synthesis (reduce the ADP/O and respiratory control ratios) [72,73]. Protonophore uncouplers also have the ability to inhibit the production of reactive oxygen species by mitochondria [72,74,75,76]. These and similar compounds are able to cross the phospholipid membrane in both protonated and anionic form due to the presence of a delocalized negative charge. Thus, they carry out cyclic transport of protons through the membrane to a compartment with a lower concentration [68,71,72,74]. In recent years, interest in protonophore uncouplers has increased significantly due to their possible use as pharmacological agents [69,70,71,76,77].

Perhaps the most fully studied natural protonophore uncouplers of oxidative phosphorylation are free monocarboxylic fatty acids [68,75,76,77,78,79,80]. It has been established that carrier proteins of the inner mitochondrial membrane that carry out the exchange transport of ADP to ATP (ADP/ATP-antiporter) are involved in the uncoupling effect of monocarboxylic fatty acids in mitochondria of vital organs of mammals (heart, liver, kidneys, skeletal muscles) [72,78,80,81,82,83]. The specific inhibitor of this carrier, carboxyatractylate, significantly reduces the uncoupling effect of fatty acids. In liver mitochondria, in addition to the ADP/ATP antiporter, the carrier that exchanges aspartate for glutamate (aspartate/glutamate antiporter) also participates in the uncoupling action of fatty acids [72,83]. It is assumed that the participation of ADP/ATP- and aspartate/glutamate antiporters in the uncoupling action of fatty acids consists in the transfer of the fatty acid anion from the inner monolayer of the membrane to the outer one, where these anions are protonated and move in the opposite direction without the participation of proteins by the flip-flop mechanism, releasing protons to the matrix [72,78,84].

The protonophore effect of bile acids was studied both on model membrane systems [19] and on isolated mitochondria [42,45,85]. Hamilton’s laboratory [19] studied the permeability of membranes for fatty and bile acids in experiments with liposomes loaded with the fluorescent pH indicator pyranine. It was found that after the addition of bile acids—CA, DCA, CDCA—to a suspension of liposomes, their neutral molecules are able to move from the outer monolayer of the membrane to the inner one (flip-flop). After reaching the inner monolayer, some of the bile acid molecules donate protons to the buffer containing pyranine, which is accompanied by a decrease in pH from 7.4 to 7.1 [19]. It has been shown that the ability of bile acids to carry protons across the membrane of liposomes depends on the hydrophobicity of their molecules, since the most hydrophilic CA is less effective among the bile acids listed above. At the same time, taurine conjugates of all three bile acids, being strong acids, are ineffective [19].

Non-conjugated bile acids (LCA, CDCA, DCA) are considered as effective uncouplers of oxidative phosphorylation [42]. These bile acids have been shown to stimulate respiration in state 4 and reduce respiratory control and membrane potential (Δψ) of succinate-fueled liver mitochondria. The most hydrophobic LCA showed the strongest effect. The less hydrophobic chenodeoxycholic and deoxycholic acids were less effective. It should be noted that the hydrophobicity of UDCA is the same as that of CDCA and DCA (Table 1), but its effectiveness as an uncoupler of oxidative phosphorylation is significantly inferior to these two bile acids [42]. Hydrophilic taurine and glycine conjugates of these bile acids were significantly less effective [42] despite their lower CMC (Table 1). The authors of this work assessed the protonophore action of bile acids by the intensity of mitochondrial swelling in an isotonic ammonium nitrate solution. It is known that the induction of proton conductivity with simultaneous transport of NH_3_ and NO_3_^−^ leads to the accumulation of osmotically active NH_4_^+^, NO_3_^−^ ions in the matrix and, as a result, swelling of mitochondria [86]. This method allows the determination of the presence of proton transport from the intermembrane space to the matrix, as is typical for passive leakage of protons through the inner membrane of energized mitochondria. At the same time, it is known that the transport of such anions as NO_3_^−^, SCN^−^, etc. through the inner mitochondrial membrane is carried out with the participation of an anion channel, whose activity can be affected by hydrophobic compounds [87]. Therefore, the swelling of mitochondria induced by bile acids observed by Rolo and colleagues [42] cannot be considered as evidence of their protonophore action.

Previously, the laboratory of V.P. Skulachev [85] found that CA, like palmitic acid and dodecyl sulfate, stimulates respiration and H^+^ conduction of the inner membrane of liver mitochondria, and this effect is suppressed by the ADP/ATP antiporter inhibitor carboxyatractylate. The authors of this work evaluated the protonophore action of these compounds in experiments on de-energized mitochondria by recording the ΔpH dissipation rate (difference in H^+^ concentrations on the inner mitochondrial membrane) using a pH meter after the rapid addition of a certain amount of hydrochloric acid. In this case, the addition of CA led to an increase in the rate of H^+^ transport into the mitochondrial matrix, as is typical for protonophore uncouplers [85]. The data obtained were considered as evidence that the ADP/ATP antiporter is involved in the uncoupling action of bile acid [84]. Supporting this suggestion is the fact that bile acids can interact directly with the ADP/ATP antiporter, as shown in proteoliposomes [88].

**Figure 2:**
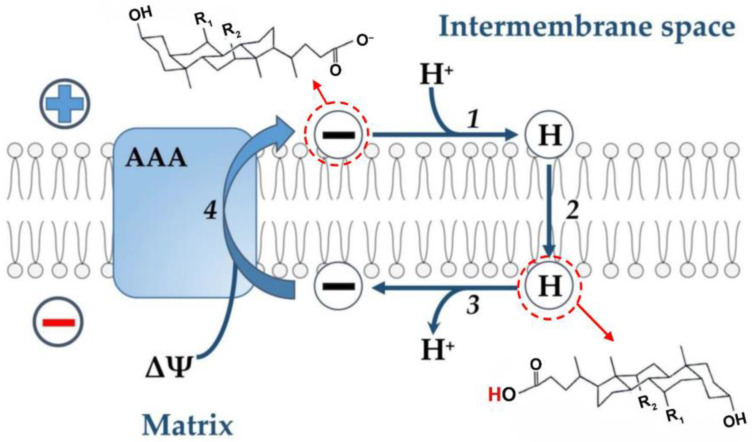
Hypothetical scheme illustrating the transport of protons by bile acid across the inner membrane of liver mitochondria with the participation of the ADP/ATP-antiporter (AAA). According to this scheme, the bile acid anion (symbol (−)) is protonated on the outer surface of the inner membrane (step 1). The neutral bile acid molecule (symbol (H)) is transported across the phospholipid bilayer to the opposite side of the membrane by the flip-flop mechanism (step 2). This stage is fast [19] and does not require any carrier. During this stage, protons are transported from a more acidic compartment (membrane space) to a more alkaline one (matrix). On the inner surface of the inner membrane, a neutral bile acid molecule releases a proton into the matrix (deprotonates) to form a bile acid anion (step 3). The bile acid anion moves in the opposite direction with the participation of the ADP/ATP-antiporter (step 4). This step 4 is electrogenic since energy in the form of ΔΨ is expended to move the anion.

Based on these data and by analogy with the uncoupling effect of fatty acids (see above), the following hypothetical scheme can be proposed (Figure 2). It is assumed that free (non-conjugated) anions are protonated on the outer surface of the inner mitochondrial membrane, since their pK_a_ on the membrane surface is 2.5 units higher than in an aqueous solution [58] and, therefore, its value is 7.5. Further, neutral molecules of bile acids are transported through the phospholipid bilayer to the opposite side of the membrane by the flip-flop mechanism, followed by the release of a proton into the mitochondrial matrix. The transport of the bile acid anion in the opposite direction is carried out with the assistance of the ADP/ATP antiporter. At the same time, it cannot be ruled out that the effects of bile acids described above may be due to damage to the inner mitochondrial membrane, as was suggested for the nonionic detergent Triton X-100 and the cationic detergent cetyltrimethylammonium bromide [85].

### 3.2. Ionophore Action of Bile Acids

It is well known that many bile acids have a relatively high affinity for Ca^2+^ [62,63,65,89,90,91]. High concentrations of Ca^2+^ (2.5–5 mM) and bile acids (2–5 mM) are able to form micellar aggregates, gels, and precipitates in aqueous solutions [62,63,65]. One should note the recent paper by Du et al., which demonstrates that bile acids can aggregate at a concentration much lower than CMC, when specific conditions are created, such as the interactions with polyelectrolytes [92]

In experiments on model lipid membranes (black lipid membrane), it is well established that free (non-conjugated) bile acids—CA and DCA—are capable of transporting divalent metal ions (Ba^2+^, Ca^2+^, Sr^2+^, Mg^2+^, and Mn^2+^) [20]. Similar results were obtained in the study of the effect of bile acids on the permeability of liposomes to divalent metal ions [21,22]. Thus, these bile acids can be considered as ionophores capable of transporting divalent metal ions, including calcium ions, directly through the phospholipid bilayer. Taurine conjugates of bile acids also have calcium ionophore activity [89]. It has been shown that bile acids increase calcium transport into red blood cells [93] and pig jejunal brush border membranes [94] while increasing Ca^2+^ concentration in kidney cell lines [95] and hepatocytes [96]. The ionophoric properties of bile acids are related to the hydrophobicity of the steroid nucleus. Thus, the ability of bile acids to raise Ca^2+^ concentration increases with increasing hydrophobicity [96]. At the same time, this action of bile acids may be associated with their effect on special carriers that carry out transmembrane Ca^2+^ transport [97].

Currently, several structures are known that transport Ca^2+^ in mitochondria. Among them, the most important are the mitochondrial Ca^2+^ uniporter (MCU), Ca^2+^/H^+^ antiporter (Letm1), Ca^2+^/Na^+^ antiporter (NCLX), and others (see reviews [40,98]. The effect of bile acids as inducers of Ca^2+^ release from the matrix was studied in experiments on isolated rat liver mitochondria [47]. In these studies, mitochondria in the presence of cyclosporin A (a Ca^2+^-dependent pore blocker, see below) were fueled by succinate, loaded with Ca^2+^, and deenergized with malonate after addition of ruthenium red, a calcium uniporter inhibitor. It has been shown that under these conditions, bile acids—LCA, HDCA, CA, and UDCA—induce the release of Ca^2+^ from the mitochondrial matrix. The release of these ions was not associated with damage to the inner membrane of mitochondria by bile acids, as it is accompanied by the generation of Δψ—the formation of a diffusion potential. It was suggested that by ejecting Ca^2+^ from the matrix, bile acids transport H^+^ in the opposite direction, i.e., carry out electrically neutral Ca^2+^/2H^+^ exchange [47].

**Figure 3:**
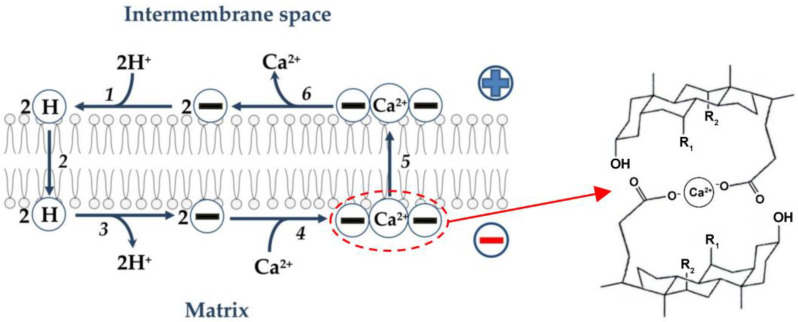
Hypothetical scheme illustrating Ca^2+^ transport by bile acids directly across the phospholipid bilayer of the inner membrane of liver mitochondria. In this scheme, the first three stages are similar to the first three stages of proton transport by bile acids, as shown in Figure 2. Two bile acid anions (symbol 2(−)) are protonated on the outer surface of the inner membrane (step 1). Two neutral bile acid molecules (symbol 2(H)) are transported across the phospholipid bilayer to the opposite side of the membrane by the flip-flop mechanism (step 2). Two neutral bile acid molecules release two protons into the matrix (deprotonate) on the inner surface of the inner membrane to form two bile acid anions (step 3). These anions interact with Ca^2+^ to form an electrically neutral complex (symbol (−)(Ca^2+^)(−)) (step 4). This complex (a hypothetical structure is depicted on the right side [89]) moves through the phospholipid bilayer of the inner membrane to its outer surface (step 5), where this complex decomposes with the release of Ca^2+^ into the intermembrane space and the formation of two bile acid ions (step 6).

As noted above, bile acids are relatively flat and rigid molecules with a polar and a hydrophobic face [1,13,16,60]. A complex can be envisioned that would consist of two such molecules, with Ca^2+^ sequestered between the two hydrophilic surfaces [89]. It has been suggested that bile acids are likely to function as mobile (in contrast to channel-forming) Ca^2+^ ionophores, with the 2:1 bile acid/Ca^2+^ complex as the possible transport intermediate [22,89]. Based on the foregoing, the following hypothetical scheme can be assumed (Figure 3). As noted above (see also Figure 2), anions of free (non-conjugated) bile acids are protonated on the outer surface of the inner mitochondrial membrane. Further, neutral molecules of bile acids are transported through the phospholipid bilayer to the opposite side of the membrane by the flip-flop mechanism, followed by the release of protons into the mitochondrial matrix. It can be assumed that a neutral complex of calcium cation with two anions of bile acids is formed on the outer surface of the inner membrane, which diffuses to the opposite side of the membrane. However, it cannot be ruled out that bile acids directly affect the Ca^2+^/H^+^ antiporter, thus stimulating Ca^2+^/2H^+^ exchange.

### 3.3. Bile Acids as Inducers of the Ca^2+^-Dependent Cyclosporine A-Sensitive Pore in the Inner Mitochondrial Membrane

As noted in the introduction, one of the links in cell death associated with mitochondria is the opening of a Ca^2+^-dependent MPT-pore in the inner membrane of these organelles for ions and hydrophilic substances, whose mass does not exceed 1.5 kDa along their concentration gradient. A highly selective inhibitor of this pore is cyclosporin A (CsA) which completely inhibits MPT-pore opening at submicromolar concentrations (see reviews [36,37,38,39,40,41]). The opening of the MPT-pore promotes rapid transfer of protons into mitochondria leading to depolarization of the inner membrane, uncoupling of oxidative phosphorylation, and, simultaneously, rapid release of Ca^2+^ from the matrix. In this case, water with dissolved low molecular weight substances rushes into the mitochondria due to colloid osmotic pressure leading to high-amplitude swelling of the organelles. Swelling of mitochondria leads to a decrease in the light scattering through the suspension, and this can be registered as a decrease in the optical density of the mitochondrial suspension [99,100]. Thus, registration of a decrease in the optical density of a suspension of mitochondria associated with the swelling of these organelles is one of the main methods for determining the pore induction [42,46,47,48,88,99,100,101].

The process of closing–opening of the CsA-sensitive pore is regulated by a number of physiological modulators. Among low molecular weight pore modulators, inorganic phosphate (P_i_) occupies a special place. It is well known that Pi increases the sensitivity of mitochondria to Ca^2+^ as a pore inductor. Pi has also been found to enhance the effect of CsA as a pore blocker [102,103,104,105,106].

It should be noted that a number of studies allows the consideration of the mitochondrial pore as a mechanism for the release of Ca^2+^ from organelles [107,108]. The question is being discussed of whether the non-selectivity of the mitochondrial pore is an important feature that allows the rapid and efficient release of Ca^2+^ from the matrix of organelles, which suggests the physiological role of this system (see reviews [40,109]).

LCA, DCA, HDCA, UDCA, as well as their glycine and taurine conjugates, are able to induce pore opening, which is inhibited by CsA in mitochondria isolated from the liver and loaded with Ca^2+^ [42,46,47,48,88,102,110]. In these experiments, the concentration of Ca^2+^ did not exceed 50 µM, and the concentration of bile acids was less than CMC by more than an order of magnitude (see Table 1). It is of note that the action of these bile acids as inducers of the mitochondrial CsA-sensitive pore is not associated with the modulation of the effect of inorganic phosphate as a pore inducer [46]. The effectiveness of bile acids as inducers of the Ca^2+^-dependent CsA-sensitive pore depends on the hydrophobicity of their molecules. The most hydrophobic LCA is the most effective, the less hydrophobic DCA and HDCA are less effective, and the more hydrophilic CA is significantly less effective [42,46]. At the same time, UDCA, being as hydrophobic as DCA and CDCA (Table 1), is significantly inferior to them in terms of efficiency as an inducer of the Ca^2+^-dependent CsA-sensitive pore [42,46]. Glycine and taurine conjugates of these bile acids are significantly less effective as inducers of the mitochondrial CsA-sensitive pore [42,102] despite their lower CMC (Table 1).

It was found that ruthenium red by inhibiting Ca^2+^ transport into the mitochondrial matrix is able to reduce the effect of bile acids as inducers of the CsA-sensitive mitochondrial pore [47]. Obviously, this is possible only if, as mentioned above, these bile acids are able to effectively induce the release of Ca^2+^ from the matrix without violating the integrity of the inner membrane, while ruthenium red prevents the return of these ions to the matrix. It is noted that other amphiphilic compounds do not have such an effect, in particular the free fatty acids: palmitic and α,ω-hexadecanedioic (Unpublished data of E. Khoroshavina). Thus, under conditions of reduced activity of the calcium uniporter, the release of Ca^2+^ from the matrix induced by bile acids may be one of the mechanisms that reduce the effectiveness of their action as inducers of the Ca^2+^-dependent CsA-sensitive pore in mitochondria.

Unlike other studied bile acids, the effects of UDCA associated with the induction of the permeability of the inner membrane (swelling of mitochondria, a drop in Δψ, and the release of Ca^2+^ from the matrix) in the presence of potassium chloride in the incubation medium, but without Pi, are also observed in the presence of CsA [46]. Obviously, these effects of UDCA, in contrast to the effects of other studied bile acids, are due to a different spatial orientation of the hydroxyl group at the seventh carbon atom of the steroid nucleus—the β-position instead of α-position, as in CDCA (Table 1). We suggest that the induction of CsA-insensitive inner membrane permeability by UDCA is associated with the activation of electrophoretic transport of K^+^ into the matrix of Ca^2+^-loaded mitochondria. This is known to be accompanied by their swelling and decrease in Δψ (see reviews [111,112]). Thus, UDCA can be considered as a K^+^ ionophore. At the same time, the involvement of other potassium ion transport systems, in particular Ca^2+^-activated K^+^ channels, cannot be ruled out [112].

## 4. Conclusions

Currently, bile acids are largely attracting attention as signaling molecules involved in the regulation of various pathways of cell metabolism by interacting with special receptors on membranes (see reviews [2,5,6,7,8,9]). Bile acids, being amphiphilic compounds, are also able to directly interact with the lipid component of membranes. Interacting with membranes, bile acids can significantly change their structure and functions (detergent action) [14,15,16,17], as well as induce transmembrane transport of protons (protonophore action) [18,19] and divalent ions (ionophore action) [20,21,22]. At the same time, further studies are needed to elucidate the mechanisms of the protonophore and ionophore effects of bile acids in the mitochondria of animals and humans. It is necessary to elucidate the dependence of these effects of bile acids on their physicochemical properties: the degree of hydrophobicity, the critical micelle concentration, the number and orientation of the hydroxyl groups of the steroid nucleus. The foregoing fully applies to the study of the mechanism of action of bile acids as inducers of Ca^2+^-dependent CsA-sensitive permeabilization of the inner mitochondrial membrane. In particular, a promising direction seems to be research aimed at elucidating the relationship between the Ca^2+^-ionophore effect of bile acids and their action as potential modulators of nonspecific permeability of the inner mitochondrial membrane and, as a consequence, cell death. As noted above, protonophore uncouplers are able to suppress the formation of reactive oxygen species in mitochondria [70,74,75,76]. It is not known whether free (non-conjugated) bile acids have a similar effect. Unlike other bile acids, UDCA is used as a drug in the treatment of liver diseases and some other pathologies [31,32,33]. Studies on intact mitochondria have shown that UDCA can be considered as a K^+^-ionophore [46]. It is known that various pharmacological agents that activate the transport of potassium ions into the mitochondrial matrix have a cytoprotective effect [113,114]. A promising line of research seems to be elucidation of the relationship between the K^+^-ionophore action of UDCA on mitochondria and its therapeutic effects.

## Figures and Tables

**Table 1:** Physico-chemical properties of the bile acids.

Bile Acid (Trivial Name)	Symbol	Position and Orientation of Hydroxyls	CMC (mM)		logP_HA_	logP_A_	logP*
			Water	0.15 M Na^+^			
Cholic acid	CA	3α*7*α12α	13	11	2.02	1.10	2.02
Taurocholic acid	TCA	3α*7*α12α	10	6	-	−0.50	0.66
Glycocholic acid	GCA	3α*7*α12α	12	10	1.65	−0.40	1.65
Chenodeoxycholic acid	CDCA	3α*7*α	9	4	3.28	2.25	3.08
Glycochenodeoxycholic acid	GCDCA	3α*7*α	6	2	2.12	0.45	2.43
Ursodeoxycholic acid	UDCA	3α*7*β	19	7	3.00	2.20	3.08
Glycoursodeoxycholic acid	GUDCA	3α*7*β	12	4	2.02	0.20	2.43
Deoxycholic acid	DCA	3α12α	10	3	3.50	2.65	3.50
Glycodeoxycholic acid	GDCA	3α12α	6	2	2.25	0.80	2.25
Lithocholic acid	LCA	3α	0.9	0.5	-	-	4.42

CMC—critical micelle concentration [52]. logP_HA_—logarithm of the octanol/water partition coefficient at pH 2.0; logP_A_—logarithm of the octanol/water partition coefficient at pH 7.4 [53]. logP*—logarithm of the octanol/water partition coefficient theoretically calculated from the structural formulas of bile acids (Computed by XLogP3 (v3.2.2 [54]).

## Data Availability

Not applicable.
